# Acetylene-Based Materials in Organic Photovoltaics

**DOI:** 10.3390/ijms11041471

**Published:** 2010-04-08

**Authors:** Fabio Silvestri, Assunta Marrocchi

**Affiliations:** 1 Department of Materials Science, University of Milan–Bicocca, via Cozzi, 53, I-20125, Milan, Italy; E-Mail: fabio.silvestri@mater.unimib.it; 2 Department of Chemistry, University of Perugia, via Elce di Sotto, 8, I-06123 Perugia, Italy

**Keywords:** acetylenes, triple-bond, conjugated polymers, poly(arylene ethynylenes), solar cells, organic photovoltaics

## Abstract

Fossil fuel alternatives, such as solar energy, are moving to the forefront in a variety of research fields. Organic photovoltaic systems hold the promise of a lightweight, flexible, cost-effective solar energy conversion platform, which could benefit from simple solution-processing of the active layer. The discovery of semiconductive polyacetylene by Heeger *et al.* in the late 1970s was a milestone towards the use of organic materials in electronics; the development of efficient protocols for the palladium catalyzed alkynylation reactions and the new conception of steric and conformational advantages of acetylenes have been recently focused the attention on conjugated triple-bond containing systems as a promising class of semiconductors for OPVs applications. We review here the most important and representative (poly)arylacetylenes that have been used in the field. A general introduction to (poly)arylacetylenes, and the most common synthetic approaches directed toward making these materials will be firstly given. After a brief discussion on working principles and critical parameters of OPVs, we will focus on molecular arylacetylenes, (co)polymers containing triple bonds, and metallopolyyne polymers as p-type semiconductor materials. The last section will deal with hybrids in which oligomeric/polymeric structures incorporating acetylenic linkages such as phenylene ethynylenes have been attached onto C_60_, and their use as the active materials in photovoltaic devices.

## 1. Introduction

Electronic and optoelectronic devices using π-conjugated organic materials as active elements, including organic light-emitting diodes (OLEDs), organic photovoltaic devices (OPVs), organic field-effect transistors (OFETs), organic photorefractive devices and so forth, have recently attracted a great deal of attention inspired by the promise of low-cost printed electronics and the significant scientific challenges that must be overcome for this goal to be realized [1–9]. Compared to silicon or germanium, organic molecules and polymers are chemically versatile, and can be synthesized in large quantities under moderate reaction conditions. They are mechanically flexible and enable vapor- or solution phase fabrication of large area devices. Furthermore, organic semiconductors may show high absorption coefficients, which make them good chromophores for optoelectronic applications [10–14]. The discovery of electrical conductivity in polyacetylene (PA) upon doping by Heeger *et al.* [15] in the late 1970s was a milestone for the use of π-conjugated organic materials in electronics. Enormous progress has been made in their design, synthesis and studies of properties and applications since.

Among organic semiconductors, two classes of materials are the most commonly used: poly-arylenes, *i.e.*, polymers consisting in aromatic rings directly connected to each other, and poly-arylenevinylenes, *i.e.*, polymers made by alternating aryl and ethylene units. Other classes of organic semiconductors, including oligo-thiophenes, metal phthalocyanines, (hetero)acenes, polytriphenylamines, perylenes, and fullerenes, have also been extensively investigated [16–22].

In the past few decades, increasing interest has focused on conjugated triple-bond containing systems, (poly)arylene ethynylenes (also referred to as (poly)arylacetylenes), as a promising class of semiconductors, due to the availability of efficient protocols for palladium catalyzed alkynylation reactions and the new conception of their steric and conformational advantages.

The development of triple-bond based materials was not correlated with double-bond based ones, probably due to an inaccurate perception of their inferior electronic properties as well as the lack of efficient approaches to their synthesis. Indeed, if we consider the most common used polymers, the delocalization and charge stabilization are better in systems as (poly)arylenevinylenes and polyarylenes than (poly)aryleneethynylenes. Additionally, larger band gaps and narrower bandwidths are generally found for poly(phenylene ethynylene) (PPE) than for its structurally close relative (poly)phenylenevinylene (PPV), which results into more difficult charge separation in (poly)arylacetylenes [23]. Despite these findings, an alkyne linkage is more accommodating than an alkene to steric and conformational constraints. For instance, steric interactions between the aromatic nuclei and the alkene units in poly(arylene vinylene)s may result in non-planar conformations, thus producing a dramatic reduction in delocalization. In contrast, direct steric interaction with alkynes may result in a bending distortion, but conjugation will be maintained due to their cylindrical electronic symmetry. Acetylene derivatives are also of interest from the standpoint of their shape-persistent rigid rod-like structures [24].

After a brief overview of the most commonly used synthetic methods leading to (poly)aryl-acetylenes, we will summarize in this review article the recent developments in their use as semiconductor materials for organic/hybrid photovoltaic devices.

## 2. Synthesis

From a synthetic point of view, remarkable advances have been made in the area of acetylenic materials in recent decades [25–31]. This impressive development and interest in the acetylene chemistry have been focused the attention on conjugated triple-bond containing systems as a promising class of semiconductors.

Palladium-catalyzed coupling reactions between aryl- or vinyl halides or triflates and terminal alkynes have led to improvements in both the selectivity and reliability of acetylenic homo- and heterocouplings, and paved the way for their application to ever more complicated systems.

**Scheme 1.**
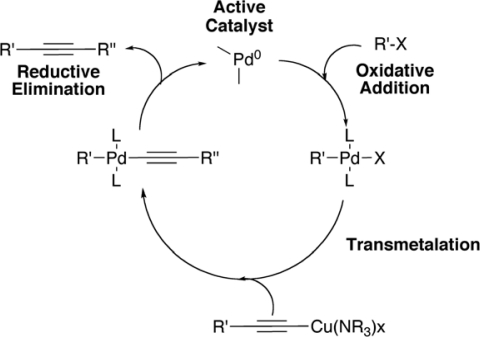
Postulated Mechanism of the Copper-Cocatalyzed Sonogashira Reaction.

In particular, the Sonogashira-Hagihara protocol (more often simply known as Sonogashira coupling, Scheme 1) has been known since 1975 and it is one of the most frequently used carbon-carbon bond forming processes in organic synthesis [32–34]. Although aryl and vinyl chlorides, bromides, and triflate are competent coupling partners, the reaction is particularly facile when using iodide substrates. As a consequence, it can be conducted under mild conditions and, in case of polymers, it can minimize cross-linking and defects formation.

**Scheme 2.**
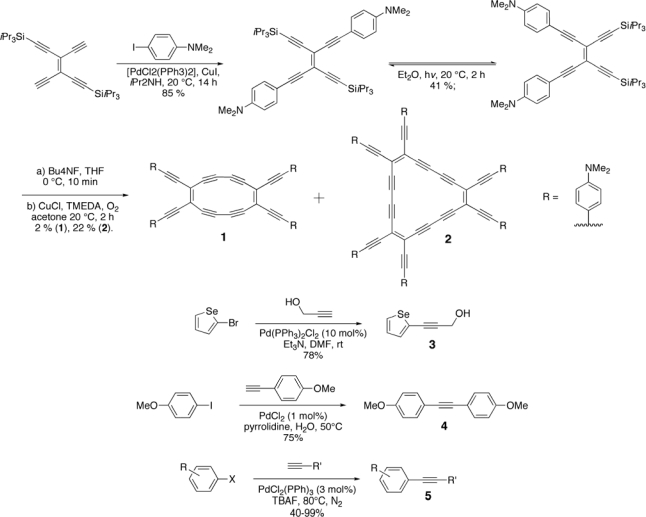
Coupling of Terminal Diynes (Glaser-Hay Coupling), and Copper Promoted/Copper Free Sonogashira Coupling of Arylenediynes with Arylhalides.

The Sonogashira reaction is generally carried out in organic solvents such as benzene, toluene, THF or dioxane. This reaction needs a base, which is usually an amine such as triethylamine, diethylamine or diisopropylethylamine. The most widely used catalysts are (Ph_3_P)_2_PdCl_2_ or Pd(PPh_3_)_4_ in conjunction with copper(I) salts, which act as co-catalysts. Several important modifications have simplified the Sonogashira reaction protocol [35–39], particularly the elimination of the copper(I) co-catalyst [40–42], since it could induce a homocoupling reaction of terminal alkynes to diynes in the presence of oxygen (Glaser-type reaction). Copper-free approaches to the Sonogashira reactions usually include the use of an amine, such as triethylamine or pyperidine, as solvent or in large excess. Recently, some copper- and amine-free methodologies have also been reported [43–46]. More recently, Fulmer *et al* [47] reported on the solvent-free Sonogashira reaction utilizing high speed ball milling. Under these conditions, iodo- and bromo- aromatics make very good coupling partners. The authors investigated the reaction under aerobic conditions. Conducting the reaction in absence of copper iodide led to moderate yields of the coupling product. However, substituting the copper iodide and conducting the reaction using a copper ball and a copper vial allows yields similar to the use of copper iodide. Selected illustrative examples highlighting the potential of palladium-catalyzed coupling reactions for generating structurally different acetylene-derived materials are shown in Scheme 2 [40,43,48,49].

**Scheme 3.**
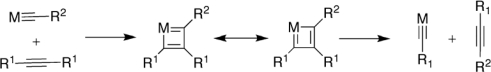
Alkyne Metathesis and Its Mechanism. The Equation Interprets the Reaction as Proceeding through the Intermediacy of Metal Carbynes.

A second additional significant synthetic method makes use of alkyne (cross-)metathesis (Scheme 3). Recently it has been shown [50–56] that this transformation hold great synthetic promise in different technological field, as molecular electronics and photonics.

**Scheme 4.**
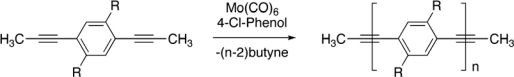
Acyclic Diyne Methathesis of Dipropynylated Benzenes by the Mortreux-Mori-Bunz Catalyst.

Alkyne metathesis refers to the exchange of alkylidyne units between a pair of (non terminal) aliphatic as well as aromatic acetylene derivatives. The generally accepted mechanism is a chain mechanism, involving the intervention of a metal-carbyne complex and a four-membered ring containing a metal [53]. The catalysts that have been most widely used, either homogeneous or heterogeneous, are based on molybdenum or tungsten complexes, in which alkoxide or phenoxide ligands are most common. For instance, the Mortreux-Mori-Bunz catalyst [54] generated *in situ* from commercially available Mo(CO)_6_ and phenol reagents was used to initiate acyclic metathesis polymerization of a series of dipropynylated benzenes to furnish polyphenylene ethynylenes in quantitative yield and high purity (Scheme 4). A promising catalytic system has been developed by Fürstner *et al* [55,56] using molybdenum complexes of the general type Mo[N(tBu)(Ar)]_3_ (Ar = 3,5-dimethylphenyl) as precatalysts that are activated *in situ* by dichloromethane. This particular reagent combination enables highly selective and efficient reactions and tolerates polar functionalites as diverse as ether, nitrile, acetals, sulfone, ketone, and silyl ether.

## 3. Photovoltaic Studies

Within the organic PVs field, there are multiple approaches: single layer diodes [57–59] organic cells based on a heterojunction between polymeric or small molecular weight materials [60–63], and organic/inorganic hybrid cells [64–67].

**Figure 1.**
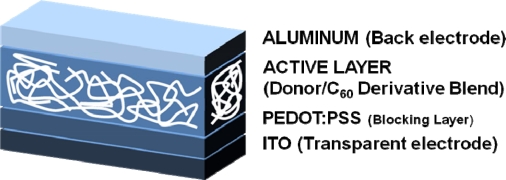
General Structure of Bulk Heterojunction (BHJ) Device.

Photovoltaic cells made with a single polymer/small molecule and two electrodes tend to be inefficient because the photo-generated excitons are usually not split by the built-in electric field, which arises from differences in the electrode work functions. The cell efficiency can be increased by splitting the excitons at the interface between an electron donor, a p-type semiconductor, and an electron acceptor, an n-type semiconductor (so-called heterojunction). Ideally, bulk heterojunction (BHJ) structures (Figure 1) represent the case of a bicontinuous composite of donor and acceptor phases, thereby maximizing the interfacial area between these two components.

It is possible to promote efficient exciton splitting and charge transport and to reduce the band gap of the p-type polymer/small molecule to absorb a larger fraction of the solar spectrum.

The device power conversion efficiency (PCE) is defined by Equation 1, where *V_oc_* is the open circuit voltage, *J_sc_* is the short circuit current (mA/cm^2^), FF is the fill factor, and P_0_ is the power of incident light source (mW/cm^2^):

(1) $$\eta =\left(V_{oc}J_{sc}\text{FF}\right)/{\text{P}}_{0}$$

The state-of-the-art in organic photovoltaics is currently represented by a BHJ solar cell based on the blend film of a polymer based on alternating ester substituted thieno[3,4-b]thiophene and benzodithiophene units (PTB7) with (6,6)-phenyl-C_71_-butyric acid methyl ester (C_71_-PCBM) [68], showing efficiencies over 7%. This device presents a good *V_oc_* (0.74 V), a high *J_sc_*, (14.5 mA/cm^2^) and a high FF (69%).

One of the basic photovoltaic parameters that influences overall performance of solar cells is open circuit voltage. Brabec *et al.* [69] demonstrated that the *V_oc_* in polymer/fullerene-based solar cells is strongly dependent on the difference between HOMO level of the donor and the LUMO level of the acceptor. Thus, an effective method to improve the open circuit voltage is therefore to manipulate the HOMO energy level of the polymers by modifying the chemical structure.

The other important parameter regarding organic solar cell efficiencies is *J_sc_*, short circuit current. This value is mostly determined by the light absorption ability of the material, the charge separation efficiency, and the high and balanced carrier mobility. Indeed, a critical issue in solar cells based on systems such as poly(3-hexylthiophene) (P3HT) or poly[2-methoxy-5-(3,7-dimethyloctyloxy)-1,4-phenylene]-alt-(vinylene) (MDMO-PPV) is the number of absorbed photons. The photon flux reaching the surface of the earth from the Sun occurs at a maximum of approximately 1.8 eV (700 nm); however, neither MDMO-PPV (E_gap_ = 2.2 eV) nor P3HT (E_gap_ = 1.9 eV) can effectively harvest photons from the solar spectrum. It is calculated that P3HT is only capable of absorbing about 46% of the available solar photons [70], and in the wavelength range between 350 nm and 650 nm. The limitation in the absorption is primarily due to limited spectral breadth, as conjugated polymers typically have extremely high absorption coefficients of the order of magnitude of 10^5^ cm^−1^ [71]. Developing a molecule that could capture all of the solar photons down to 1.1 eV would allow absorption of 77% of all the solar photons [72].

Within this frame, (poly)arylacetylenes are particularly attractive because they exhibit distinguishing spectro-electrochemical characteristics, resulting from the electron-withdrawing character of the triple bond, and their rigid rod-like structure. Moreover, the availability of efficient synthetic protocols allows variation of the effective π-conjugation length of these structures, as well as functionalization of their peripheries with suitable chromophoric groups. Therefore, a broad range of arylacetylenes has been synthesized and evaluated for use in OPVs, going from donor small molecules to (co)polymers to donor (oligomer or polymer)-fullerene hybrids.

### 3.1. Small Molecules

Recently, increasing attention has focused on soluble small molecules (**S**) as donors, since they offer the facile processing associated with polymers, are monodisperse, are easier to purify, and typically exhibit higher charge carrier mobilities [73,74] compared to other organic semiconductor. To date, the small molecule solar cells with the highest PCE (*i.e.*, 4.4%) have been fabricated with an active layer composed of a blend of a diketopyrrolopyrrole-containing oligothiophene (donor), and a fullerene derivative C_71_-PCBM (acceptor) [75]. This section discusses the use of molecular arylacetylenes as the active materials in OPV cells. Details on their preparation are not provided here, and the reader is referred to the original papers for greater depth and scope.

**Figure 2.**
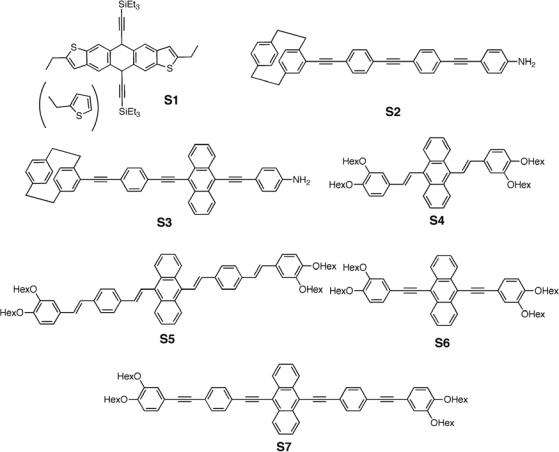
Structure of Acetylene-containing Small Molecules for Organic Photovoltaics.

Lloyd *et al.* [76] reported photovoltaic cells based on solution-processed blends using easily prepared 2,8-diethyl-5,11-bis(triethylsilylethynyl)anthradithiophene (**S1**) (Figure 2) as the donor and (6,6)-phenyl-C_61_-butyric acid methyl ester (PCBM) as the acceptor. Conjugated acene molecules have been shown to exhibit high solubility in common organic solvents and large field-effect mobilities, which may be accompanied by relatively large exciton diffusion lengths, desirable in BHJ solar cells. Solvent vapor annealing of these blends induces the formation of spherulites, which consist of a network of anthradithiophene crystallites dispersed in an amorphous matrix primarily composed of fullerene. A direct correlation between device coverage with spherulites and its performance was found, leading to a PCE of 1%.

Recent efforts in our group [77–79] focus on the synthesis and characterization of molecular arylacetylenes and their implementation in photovoltaic devices. The syntheses of the [2.2]para-cyclophane-based [80] molecular systems (**S2**, **S3**) (Figure 2) comprising three *para*-connected carbon-carbon triple bonds have been reported and their use in single layer organic solar cells has been explored [77]. Organic solar cells were fabricated with the structure of FTO/PEDOT:PSS/Active layer/LiF/Al and tested under a halogen tungsten lamp with an intensity of 76 mW/cm^2^. A maximum photocurrent quantum efficiency of 13.24% was achieved with compound **S2**; photovoltaic effect was reached with a *V_oc_* of 1.26 V, *J_sc_* of 2.3 × 10^−2^ mA/cm^2^, and a PCE of 0.008%. The overall efficiency of device was low but we were able to demonstrate that the presence of an anthracene unit in the π-conjugated side chain resulted in a larger photon harvesting, thus contributing to the preservation of a high open circuit voltage and the increase in the short circuit density.

Very recently, we reported the synthesis of new anthracene derivatives (**S4–S7**) [78,79] (Figure 2) and their implementation as donors in bulk heterojunction solar cells having PCEs of up to ∼1.2% under standard AM1.5G conditions. Each donor system was designed to achieve both high solubility in common organic solvents and an extension of the absorption spectra toward low energies to enhance solar light harvesting. Importantly, the comparative study of compounds **S4–S7** allows understanding of the effects of the replacement of the acetylenic for the olefinic π-spacer on the chromophore electronic structure, physical properties, and device efficiencies.

The OPV active layers consisted of one of compounds **S4**–**S7** as the donor and PCBM as the acceptor, blended in a 1:1 wt/wt ratio. The open-circuit voltage values for devices fabricated with olefinic compounds **S4** and **S5** were lower than those for acetylenic donors **S6** and **S7** and, interestingly, track the progression in the HOMO energy levels. The short circuit currents measured for devices based on olefinic donors **S4** and **S5** were low, affording a poor PCE, especially for **S4**/PCBM (*i.e.*, 0.04%). This is consistent with the much higher LUMO energies present in **S6** and **S7** *versus* **S4** and **S5**, which is a result of the more electron-withdrawing character of the triple bond. On the other hand, the devices fabricated with acetylenic donors **S6** and **S7** exhibited short-circuit current densities of 2.62 and 2.64 mA/cm^2^, respectively, and substantial open circuit voltages of 0.96 and 0.93 V, respectively. Combined with fill factors of 45% and 41%, these data yielded PCEs of 1.17% and 1.02%, respectively. The spectral response of the **S6**/PCBM and **S7**/PCBM based OPVs revealed an appreciable EQE response in the 400–500 nm wavelength range (∼27%). BHJ solar cells based on the acetylenic donors employed in this study were found to be significantly more efficient than those based on the olefinic donors, thus underscoring the potential of acetylenic π-donors in organic photovoltaics.

### 3.2. (Co)Polymers and Metallopolyyne Polymers

In this section, the properties and application in organic photovoltaics of (co)polymers (**P**) containing triple bonds, and metallopolyyne polymers (**MP**) are covered. For details on the synthetic methods leading to the specific systems the interested reader is referred to the original papers.

#### 3.2.1. Polymers

**Figure 3.**
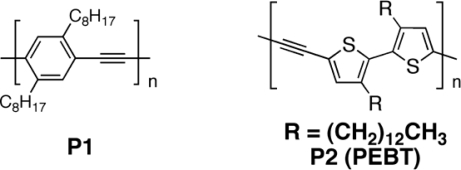
Structure of Poly-dioctyl phenylene ethynylene (PPE) and Poly(ethynylene-bithienylene) (PEBT).

Umnov and Korovyanko [81] studied a poly-dioctyl phenylene ethynylene (**P1**, Figure 3) (PPE)/C_60_ blended cell, as well as bilayer cells to evaluate the *V_oc_* dependence on excitation photon energy. The authors found that it was possible to satisfactorily describe such dependence by an energy diagram containing both exciton and free carrier pair levels, and conjectured that *V_oc_* in two-component organic photocells is correlated to the energy difference between the built-in potential and exciton binding energy in absorbing material.

Cremer *et al.* [82] synthesized a poly(ethynylene-bithienylene) (PEBT, **P2**) (Figure 3) and compared its photovoltaic behavior with P3HT. This work underlined the positive influence of triple bond on the open circuit voltage, reaching an impressive enhancement from 0.62 V to 1.03 V, one of the highest values reported in literature. The UV-vis absorption spectra of **P2** and P3HT in chloroform were very similar, with π-π* absorption bands that maximize at 451 nm and 448 nm, respectively. Apparently, introduction of ethynylene groups into the backbone of P3HT only marginally affected the optical transitions. In thin films, the absorption spectra of **P2** and P3HT exhibited partly resolved vibrational fine structure and showed a significant red shift of the π-π* transition that was attributed to planarization of the π-system and interchain interactions induced by aggregation and crystallization. The lowering of the HOMO level in **P2** by 0.3 eV as compared to P3HT was due to the electron-withdrawing character of the ethynylene units in the chain. Moreover, the red shift in the absorption spectra revealed a tendency for **P2** to form aggregated structures, albeit somewhat less than for P3HT. In contrast to P3HT, **P2** did not aggregate in mixed films with PCBM, as deduced from the lack of a red shift in the absorption spectrum in the blend. This lack of aggregation limited the number of absorbed photons and likely caused a lower mobility for the photo-generated holes. As a consequence, the short-circuit current and fill factor of these **P2**/PCBM devices were limited, reaching a maximum value of respectively 3.1 mA/cm^2^ and 36%. A PCE of 1.13% was achieved.

**Figure 4.**
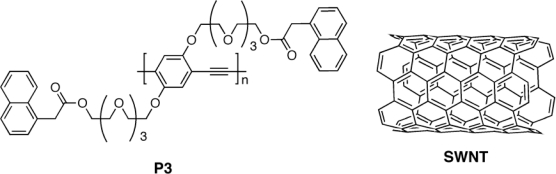
Structure of Modified Poly(phenylene ethynylene) (**P3**) and Single Wall NanoTubes (SWNT).

Very recently, Liu *et al.* [83] designed a novel bulk heterojunction structure based on a poly(phenylene ethynylene) (**P3**)/Single Wall NanoTube (SWNT) to improve the dispersion of SWNTs in the composite based on their structural similarity and strong interaction (Figure 4). Compared with C_60_, SWNTs have many advantages, including high charge mobility, long π–π conjugation and large aspect ratio. However, the advantages of using SWNTs in photovoltaic devices are somewhat hindered due to their insolubility and poor dispersion in a polymer matrix. This is because the device performance depends not only on the properties of the nano-fillers, but also on their arrangement in the thin-film.

It is known that active layer morphology plays a key role in the device fabrication; a better dispersion of SWNTs would inevitably lead to higher donor/acceptor (D/A) interfaces in the active layer, which facilitates the efficiency of exciton dissociation, and provides more conducting channels for charge transfer, thus leading to a high power conversion efficiency. The authors demonstrated a much higher open circuit voltage and a higher energy conversion efficiency relative to a control device fabricated by using a poly(3-octylthiophene) (P3OT)/SWNT active layer. Indeed, a *V_oc_* of 1.04 V and a *J_sc_* of 0.27 mA/cm^2^ were achieved. The difference in electron affinity between the donor (**P3**) and acceptor (SWNTs) originated a built-in potential that broke the symmetry, thereby providing a driving force for the dissociation of the photo-generated excitons into electrons and holes.

The overall power conversion efficiency of the **P3**/SWNTs based device was 0.05%, which was more than 2× higher relative to the P3OT- based device one (0.02%). The potential interaction between highly delocalized π-electrons of carbon nanotubes and π-electrons correlated with the lattice of the polymer skeleton allow to form an interconnecting network and provide a direct pathway for enhanced charge transport, thus inducing an increase in *J*_sc_.

#### 3.2.2. Copolymers

The copolymer approach to the development of a new material aims at combining the intrinsic characteristics of the constituting monomers with novel structure-specific properties. The introduction of electron-withdrawing acetylene units within the PPV backbone opened a way to new types of π-conjugated systems, denoted PPE-PPVs. PPE-PPVs may in principle combine the high quantum yields of fluorescence, as exhibited by PPEs, with suitability and stability, as it is found in the case of PPVs [84].

**Figure 5.**
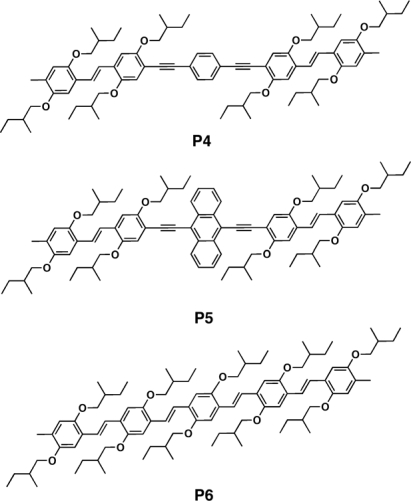
Structure of PPE-PPV Oligomers **P4–P6**.

These materials were obtained through Wittig-Horner-Emmonds reaction between an acetylene containing phosphonate and/or aldehyde precursors. To attain insight into the structural and electronic properties of such hybrid conjugated aromatic polymers, Bredas *et al.* [85] carried out experimental and theoretical investigations on a number of corresponding monodisperse conjugated oligomers. They demonstrated the tunability of the electronic properties of the materials by inserting double/triple bonds and by changing the aromatic cores within a given oligomeric backbone. By substituting the inner benzene ring (**P4**) with an anthracene unit (**P5**) or by switching from triple bonds to double bonds around the central unit (**P6**) (Figure 5), the authors also showed that it is possible to red-shift the absorption spectra. Anthracene substitution also allowed to remarkably red-shift the fluorescence [86].

**Figure 6.**
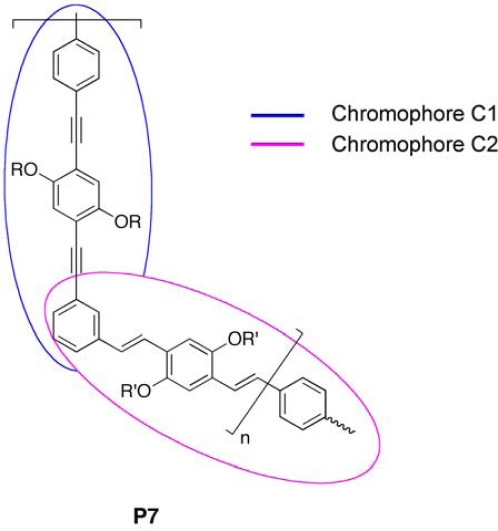
Structure of PPE-PPV Copolymer **P7**.

Chu *et al.* [87] demonstrated that hybrid systems are also interesting for the high energy transfer between PPV and PPE counterpart. They found that the absorption of the copolymer **P7** (Figure 6) corresponding to the sum of the absorption of the chromophores blocks **C1** and **C2**. However, the fluorescence spectrum exhibited emission peaks which were quite similar to those observed for the chromophore block **C2**, thus indicating an efficient energy transfer from PPE moiety to PPV counterpart. Photoexcitation dynamics and laser action studies in solution were also carried out for PPE-PPV copolymer, confirming the interchain interaction between the two moieties [88]. These results elucidated the behavior of the hybrid conjugated system as well as the effects due to the functionalization with specific moieties, playing a key role in the design of polymers with suitable features for OPV applications.

**Figure 7.**
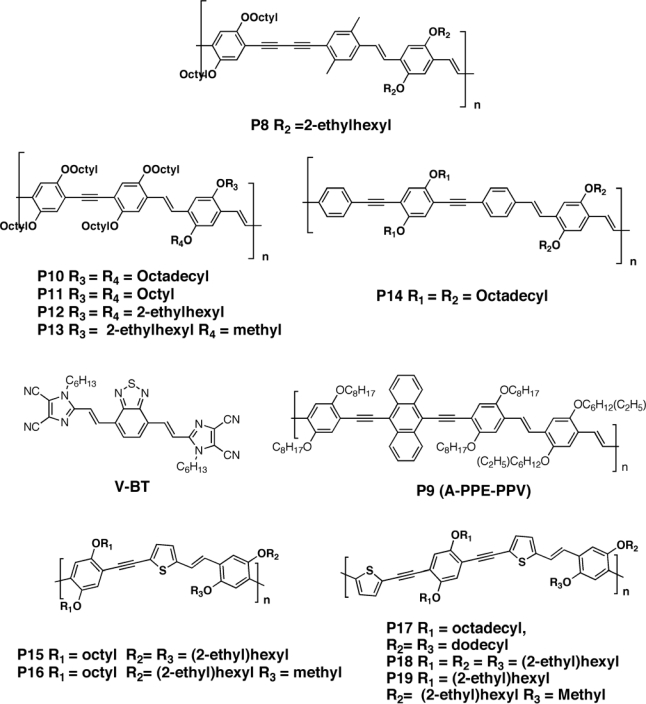
Structure of Modified PPE-PPV Copolymers **P8**–**P19**.

Egbe *et al.* [89–92] synthesized novel PPE-PPV copolymers for photovoltaic devices. In their earlier work, they reported that hybrid arylene-ethynylene/arylene-vinylene polymers **P8** and **P9** (Figure 7) exhibited a higher open circuit voltage relative to MDMO-PPV systems. The increased open circuit voltage was attributed to the electron-withdrawing nature of the triple-bond moieties, which led to an enhanced electron affinity, and consequently, to a higher oxidation potential (*i.e.*, improved oxidation stability) as well as to a lower HOMO level (*i.e.*, a higher ionization potential). Supporting the idea about the origin of the open circuit potential [69] originates from the quasi-Fermi level splitting between the donor HOMO and the acceptor LUMO, a lower donor HOMO level corresponds, in general, to an improvement in the device efficiencies. For the device made with **P9** and PCBM (1:2 wt/wt) [89], a *V_oc_* of 0.81 V was observed with a quite low short circuit current, because of the large scale phase segregation, of 4.3 mA/cm^2^ and a FF of 59%, resulting in a PCE of 2%. Through further investigations, Egbe and co-workers have found that photovoltaic devices based on **P9**/PCBM blends with a weight ratio of 1:2 [93] and 1:1 [94] showed efficiencies of 3.14% and ∼2.5%, respectively. The difference between PCE values achieved in ref. 89 and ref. 93 was ascribed by the authors to differences in polymer **P9** molecular weights as well as differences in the experimental conditions.

Molecules with similar structure (**P12**–**P14**) were studied by Al-Ibrahim *et al.* [95]; the best result was obtained with a device based on **P12** (Figure 7), which gave the following parameter values: *V_oc_* = 0.86 V, *J_sc_* = 4.2 mA/cm^2^ and FF = 46%. By using **P14** as donor material the same authors achieved a device exhibiting *V_oc_* = 0.72 V, *J_sc_* = 1.86 mA/cm^2^ and FF = 38%.

The alkoxy side chain was found to influence the devices performances [96–98]. Of particular note, the *V_oc_* value of devices based on the copolymers **P10**–**P14** investigated in the work were found to depend much more on the length and nature of the grafted alkoxy side chains than on HOMO energy levels. For example, the open circuit voltage changed from 750 mV for **P10** to 900 mV for **P14** (Figure 7). Indeed, longer side chains not only limited the interfacial area between the donor conjugated backbone and the acceptor components, but might also favor an easy recombination of the photo-generated charges by elongating the percolating path as well as hampering the transfer of charges to the electrodes. Short circuit current was also found to be affected by the insulating nature of the longer side chain with a maximum around 2 mA/cm^2^; the highest PCE value obtained was 1.75% for **P12**.

To better evaluate the efficiency of acetylene-based polymers, Sellinger and coworkers [99] compared poly(2,5-dioctyloxy-1,4-phenylene-ethynylene-9,10-anthracenylene-ethynylene-2,5-dioctyloxy-1,4-phenylene-vinylene-2,5-di(2′-ethyl)hexyloxy-1,4-phenylene-vinylene (A-PPE-PPV) (**P9)** and P3HT with a novel acceptor material, 4,7-bis(2-(1-hexyl-4,5-dicyanoimidazol-2-yl)-0vinyl)benzo[c]1,2,5-thiadiazole (V-BT). The LUMO energy level of **P9** was found to be −3.1 eV, and the LUMO of V-BT (−3.49 eV) was sufficiently low to allow photo-generated excitons on **P9** to be separated at the hetero-interface. Both the A-PPE-PPV: V-BT and PH3T:V-BT devices exhibited low efficiency (<0.4%) due to the very low J_sc_ and FF. As expected because of a sufficiently large LUMO(V-BT)- HOMO (**P9**) offset, the V_oc_ value of the corresponding device was high (∼0.9 V), whereas for P3HT-based device a V_oc_ < 0.6 V was reached. By using this acceptor, the efficiency in the P3HT-based solar cells was limited by an incomplete dissociation of the photo-generated excitons, as indicated by incomplete photoluminescence (PL) quenching. On the opposite side, **P9/**V-BT blends showed nearly complete quenching and thus very efficient exciton separation. However, missing percolation pathways and recombination via exciplex emission seemed to be the limiting processes. The low FF (30%–40%) observed for each donor polymer blended with **V-BT** was attributed to low electron mobility.

Polythiophenes, e.g., poly(3-hexylthiophene), have been proven to be efficient donor materials for photovoltaic devices in conjunction with fullerenes. Relatively high short circuit currents of up to 10 mA/cm^2^ were measured, which was attributed to a high degree of intermolecular ordering leading to high charge carrier mobility [100,101]. Egbe *et al.* [102,103] synthesized thiophene-containing arylene-ethynylene/arylene-vinylene alternating copolymers with 1:2 and 2:2 ratio of triple-bond/double- bond units and explored the BHJ device performances. By reducing the number of the triple bonds units in the polymer the photovoltaic performance was found to improve. Indeed, a higher number of triple bonds resulted in lowering the mobility of the photo-generated charges, and also enabled strong π–π interactions among the donor molecules, thereby limiting the contact area between donor and acceptor molecules. The polymer **P15** (Figure 7) reached a maximum efficiency of 1.21% while that for **P16** was 1.74%, with an increase in the short circuit current of 40% for the latter. As expected, the open circuit voltage for both **P15** and **P16** was higher than in P3HT. This was attributed to a larger donor-acceptor interfacial area in **P15-** than in **P16-**based cells, as evidenced by the smaller nanoscale clusters size observed in the AFM topology. By inserting a further thiophene ring to obtain polymer **P17**–**18**, the cell characteristics were found to be much more influenced by the alkoxy chains. The best results were obtained for **P18**-based solar cells, with an open circuit voltage of 0.8 mV, a short circuit current of 4.19 mA/cm^2^ and a PCE of 1.52%. Going from **P17** to **P19** (that differ from another only in length of the alkoxy chain) the device efficiency decreased of ∼5×. The AFM images did not show clear donor–acceptor phase separation of the active layer in **P18** and **P19** cells, thus confirming their similar photovoltaic performance. However, a very large phase separation arises after attaching long dodecyloxy and octadecyloxy side chains as in the case of **P19**. In contrast to the PPV-type polymers, PPEs having structures closer to those of the PPV, have received much less attention, probably because their absorption did not well match the solar spectrum.

**Figure 8.**
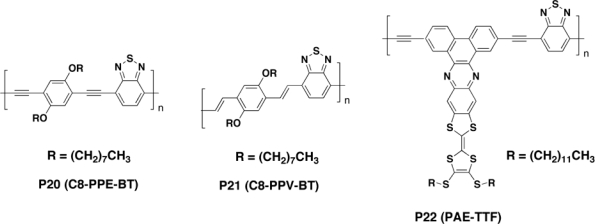
Structure of Copolymers with Donor-Acceptor Architectures.

Lu *et al.* [104] synthesized a novel PPE derivative (**P20**, Figure 8) containing benzothiadiazole (BT) co-monomer. The alternation of donor and acceptor units in the main chain of **P20** resulted in the considerable shift of the maximum absorption band position to a longer wavelength of 485 nm compared with that of pure PPE (374 nm), indicating the presence of extensive π-conjugated system. A strong charge transfer from the π* band of **P20** to PCBM was observed in the excited state but the efficiency of the resulting BHJ device was very low (0.022%). The open circuit voltage was good (0.7 V), thus confirming the ability of the triple bond to decrease the HOMO energy levels; unfortunately, the low short circuit current (82.7 μA/cm^2^) and low fill factor (0.29%) adversely affected the global efficiency of the device. Comparing the photovoltaic performance of **P20** to **P21** (Figure 8), in which triple bonds are replaced by double bonds to connect dioctyloxybenzene units and benzothiadiazole units, it was demonstrated a somewhat higher efficiency of **P21**/PCBM blend device (0.335%). This was tentatively attributed to **P21** better ability in transferring electrons to PCBM, as well as to a more effective conjugation length, leading to a broader optical absorption range [105].

Hou *et al.* [106] prepared a novel tetrathiafulvalene (TTF)-fused poly(arylene ethynylene) **P22** (PAE-TTF, Figure 8) where the linear main chain acts as electron-deficient acceptor and the π-conjugated TTF units act as electron-rich donor in the side chains. Intramolecular charge transfer between the TTF side chains and the main chain was demonstrated. The electroactivity of the TTF units was found to be closely associated with the HOMO–LUMO levels of the conjugated polymer. Effective π–π stacking in the solid state was ensured from the coplanarity of the acceptor main chain and the donor TTF side chains. A photovoltaic device based on PAE-TTF has been fabricated and characterized. Initial studies revealed that the corresponding open-circuit voltage, short-circuit current, fill factor and power conversion efficiency were 0.42 V, 2.47 mA/cm^2^, 24.2% and 0.25%, respectively. The lack of effective absorption in the red to near-infrared region of the solar spectrum was considered to account for the low power conversion efficiency. Although the power conversion efficiency was relatively low, it excels that of other reported TTF-fused systems, indicating that this kind of TTF-fused polymers might become a promising active material for photovoltaic devices.

**Figure 9.**
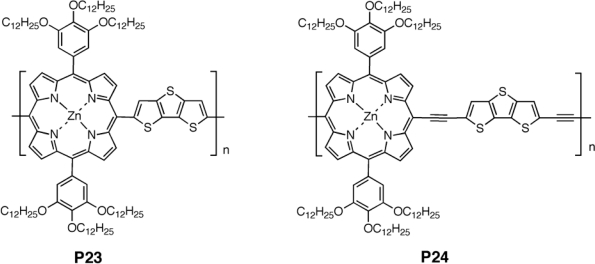
Structure of Porphyrin-Dithienothiophene π-Conjugated Copolymers **P24** and **P25**.

To ensure better solar emission spectrum coverage, it is also possible to introduce a strong chromophore like a porphyrin into the polymer skeleton, as reported by Huang *et al.* [107]. The planarity and S···S interaction of fused thiophenes in the backbone also promoted highly ordered π-stacked structures and high hole mobilities. The single bond linked polymer **P23** showed higher molecular weight and better thermal stability than the triple-bond linked counterpart **P24** (Figure 9). The Soret band and Q-band of the latter were further red-shifted by ∼28 and 94 nm, respectively; the Q-bands of **P24** were broadened and stronger, compared to that for the single-bond linked counterpart **P23**. The absorption spectrum of polymer **P23** as a thin film was similar to that obtained in solution, which was likely a result of the twisted main chain. However, the Soret band and Q-band of **P24** were broadened and red-shifted by 18 and 57 nm respectively, on going from solution to solid state. It is worth noting that the Q-band of **P24** in thin-film was located at 650–850 nm and was much stronger than that in solution. This is beneficial to sunlight harvesting, as the solar emission spectrum peaks at 600–800 nm. The red-shifted absorption and stronger Q-band of **P24** as a thin film was probably related to the aggregation caused by a more coplanar main chain. The triple-bond linked polymer **P24** was electrochemically active in the oxidation as well as the reduction region, while the single-bond linked polymer **P23** showed only an oxidation peak potential.

Field-effect hole mobilities up to 2.1 × 10^−4^ cm^2^ V^−1^ s^−1^ were obtained for these porphyrin-dithienothiophene copolymers. **P24** showed higher mobility than **P23** at room temperature, which was attributed to a more coplanar and extended π-conjugated main chain in **P24**, stronger aggregation and intermolecular interactions in the solid state. Thermal annealing of **P24** led to a different film quality and, therefore, different device performance. An optimal PCE of 0.3% was achieved using **P24**:PCBM (1:3, w/w) as the active layer, which is among the highest PCEs reported for the device based on porphyrin-containing molecules and polymers. Open-circuit voltage, short-circuit current and fill factor reached 0.58V, 1.52 mA/cm^2^, 0.34%, respectively. The PCE of the solar cell based on **P24**:PCBM (1:3, w/w) was twice as great as that based on **P23**:PCBM (1:2, w/w). This correlates well with the stronger Q-band absorption and higher mobility at room temperature observed for **P24**.

**Figure 10.**
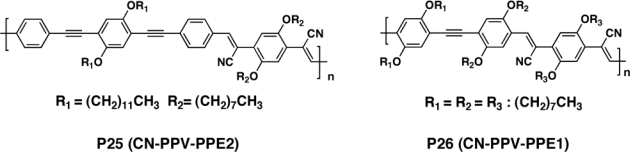
Structure of Alkyne-Containing CN-PPVs **P25** and **P26**.

Egbe *et al.* [108,109] employed alkoxy-substituted CN-containing phenylene-vinylene-phenylene-ethynylene hybrid copolymers (CN-PPE-PPV), **P25** and **P26** (Figure 10), as acceptors in single layer, bilayer and bulk heterojunction devices. The inclusion of the cyano group caused a decrease in the HOMO and LUMO energy levels, which perfectly matched donor, poly[2,5-dimethoxy-1,4-phenylene-1,2-ethenylene-2-methoxy-5-(2-ethylhexyloxy)-1,4-phenylenevinylene-1,2-ethenylene (M3EH-PPV). An interesting feature in these results was that the copolymers **P25**, whose constitutional units include two triple bonds, showed smaller discrepancy between optical and electrochemical band gap energies to that of copolymer **P26**, whose repeating unit consists of one triple bond. This was interpreted in terms of similarities and differences in the polymer chains alignment (*i.e.*, thin-film morphology) in films prepared by spin-coating for optical investigations and by drop casting for the electrochemical studies. Indeed, the presence of a higher number of triple bonds in **P26** enhanced the conjugated backbone rigidity (and coplanarity), leading to a greater intrinsic self-assembly ability, thereby limiting the dependence of the film morphology on the preparation methods.

As expected, the efficiencies for single layer cells were quite low. However, the device containing polymer with only one triple bond on the repeating unit (**P26**) showed a surprisingly high IPCE with 3.9%. On the contrary, pure single layer devices with **P25** reached only a peak IPCE of 0.15%. Solar cells based on a blend of M3EH:**P25** (1:1 wt/wt) achieved an IPCE of 26%, which is among the highest values reported for polymer-polymer blend devices. Interestingly, the open circuit voltage of these devices reached ∼1.5 V. Similar high *V_oc_* of 1.5 V and IPCE of 16% were obtained in M3EH:**P26** blend. As a result, the energy efficiencies under white light were 0.80% and 0.62% for **P25** and **P26**, respectively. The small fill factor (16%–26%) observed for the blend devices suggested that the electron mobility in these hybrid polymers was rather low. This hypothesis was supported by the photovoltaic performance of **P25** and **P26** in bilayer devices, which was found to decrease with increasing thickness of the acceptor layer. In particular, in the case of **P25** (two triple bonds), the FF dropped from ∼23% from the thinnest acceptor layer to less than 13% for the thickest, whereas it dropped to ∼18% for compound **P25** (one triple bond). The increasing number of ethynylene units into the backbone of CN-PPV seemed therefore to reduce the ability to transport electrons. The authors rationalized this finding in terms of a low rotational barrier around the triple bond, which increases the energetic disorder of single polymer chains. The *V_oc_* of the bilayer devices from **P25** and **P26** was ∼1.5 V. However, because of the small fill factor and the low IPCE, the energy efficiency was only 0.6% in both cases.

**Figure 11.**
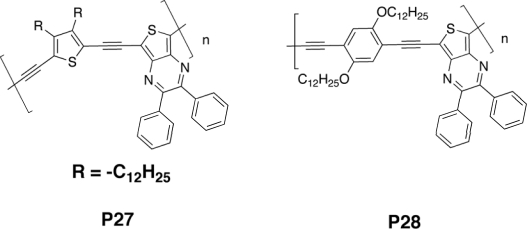
Structure of Poly(heteroaryleneethynylene)Copolymers **P27** and **P28**.

Ashraf *et al.* [110] synthesized two poly(heteroaryleneethynylene) derivatives containing thieno[3,4-b]pyrazine as the acceptor and thiophene (**P27**) or dialkoxyphenylene (**P28**) as the donor, respectively, to achieve absorption in the 390–800 nm region (Figure 11). Both materials have low optical band-gaps (∼1.57 eV), due to donor-acceptor push-pull effects through the conjugated triple bonds. Photovoltaic devices based on the blends of **P27**, **P28** (as electron donor) and PCBM (as electron acceptor) has been fabricated on a polyester foil. The cell based on **P27**/PCBM (1:1 wt/wt) showed an open circuit voltage of 0.67V, a short circuit current of 10.72 mA/cm^2^, and a PCE value of 2.37%. The corresponding device parameters found using **P28**/PCBM (1:2 wt/wt) were 0.7 V, 4.45 mA/cm^2^, and 1.36%.

#### 3.2.3. Metallopolyyne Polymers

Inclusion of transition metals into macromolecular organic scaffolds allows the hybridization of the interesting physical characteristics of metal complexes such as electronic, optical and magnetic properties with the solubility and processability inherent to carbon-based polymers. Within the framework of synthetic metal-containing polymers, rigid-rod transition metal-acetylide polymers, or metallopolyynes (**MP**) in short, have spurred tremendous global interest at the forefront of many organometallic polymer investigations.

The interest in these materials, and in platinum polyynes in particular, lies in the nature of interaction between the conjugated ligands and the metal. Pt-polyyne is a metal complex in a square planar configuration. The complexation of an electron-donating transition metal (Pt) ion into the polymer main chain was reported to enhance the intrachain charge transport of π-conjugated polymers [100,101,111,112]. When Pt metal is conjugated with an alkyne unit, the *d*-orbitals (*d_xy_* and *d_xz_*) of the Pt overlaps with the π-orbitals (π_y_* and π_z_*) of the alkyne unit leading to an enhancement of π-electron delocalization along the polymer chain. The metal ions act as a barrier to this delocalization, so that optical excitations are expected to have the character of molecular excited states and thus be in the form of strongly bound excitons. Most of the photo-excited states in these organometallic polymers are triplet excitons. Indeed, the triplet exciton is confined to one monomer unit [113]. With reference to the Jablonski energy level diagram in a simple photoluminescence (PL) system, there are two radiative decay processes upon the absorption of photons by a molecule from the ground state (S0), fluorescence (Sz→S0) and phosphorescence (T1→S0). The relative positions of the lowest singlet (S1) and triplet (T1) excited states strongly affect the intersystem crossing (ISC) rate into the triplet manifold. This provides a major non-radiative decay mechanism for organic systems, thereby reducing the PL efficiency in purely fluorescent molecules. Triplet states are not produced efficiently as a result of a direct excitation of most organic conjugated materials. Therefore it is necessary to use materials that incorporate heavy atoms, which give rise to efficient intersystem crossing by enhancing the spin-orbit coupling. Platinum acetylide polymers represent a class of π-conjugated materials featuring high quantum efficiency for intersystem crossing (to produce the triplet excited state) following direct excitation.

It is thought that triplet excitations play an important role in optical and electrical processes within conjugated organic polymers with direct implications for their technological applications in optoelectronics and photonics [114]. Indeed, triplet excited states can be harnessed to increase the efficiency of charge generation in OPV active materials [115,116]. The long lifetime of the triplet state may enhance the probability of exciton diffusion to a donor-acceptor interface. What’s more, quantum mechanical spin restrictions prevent charge recombination in the geminate ion-radical pair produced as a result of photoinduced charge transfer from a triplet state precursor.

Despite this fact, metallated conjugated polymers have rarely been tested as materials for organic solar cells. We focused this part of the review on soluble π-conjugated organometallic poly-yne polymers of the form trans-[-Pt(PBu_3_)_2_C≡CRC≡C-]_n_ (R = arylene or heteroarylene). These systems are mainly obtained by the coupling reaction between bis-terminal alkyne units and *trans*-dichlorobis(trialkylphosphine) platinum(II) unit through dehydrohalogenation methods [117,118].

**Figure 12.**
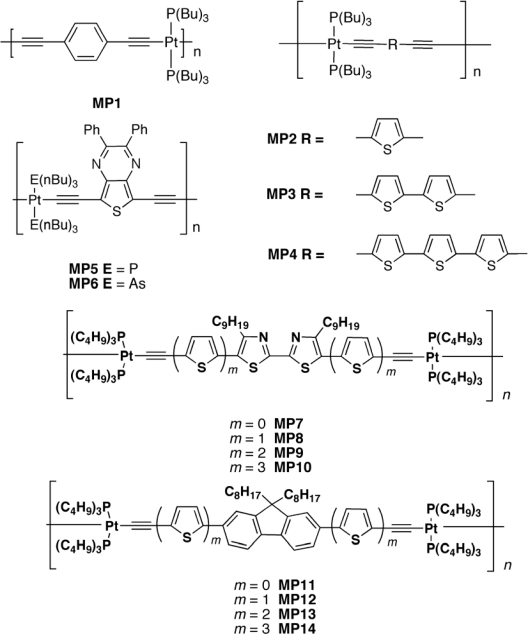
Structures of Metallopolyyne Polymers **MP1–MP14**.

The prototype for much of the initial metallopolyyne work was the Pt poly-yne **MP1** (Figure 12). Köhler *et al.* [119] showed that the maximum photocurrent quantum efficiencies for carrier generation in single-layer neat polymer **MP1** cells of ITO/**MP1**/Al were ∼0.03–0.6%, and the performance was comparable to that found in similar devices made with PPV. Remarkably, the quantum efficiency of ∼1–2% could be achieved for the ITO/**MP1**:C_60_/Al cell with the addition of 7 wt% of C_60_ [113]. They provided evidence that the polymer triplet state was active in charge generation by observing that C_60_ only partially quenched the polymer’s singlet emission but completely quenched the triplet emission.

In 1999 Chawdhury *et al.* [120] demonstrated the same using higher conjugated polymers with thiophenyl-, dithiophenyl- or terthiophenyl chain between bridging triple bonds (Figure 12). With increasing thiophene content for the oligothienyl chain the optical gap value decreased; this was attributed to a greater delocalization of π-electrons along the polymer backbone. It was also noted that when the number of thiophene units increased, the overall effect on the band gap decreased. From the photocurrent action spectra of the Au/**MP2**/Al, ITO/**MP3**/Al, and ITO/**MP4**/Al photocells, polymers appeared to be good photoconductors and showed a photocurrent quantum efficiency of ∼0.04% at the first photocurrent peak, which is common for many single-layer devices. The photoconducting properties did not depend on the size of the thiophene fragment.

More recently, an external quantum efficiency of ∼9% has been achieved by Guo *et al.* [121] for the ITO/PEDOT-PSS/(**MP2**)-PCBM(1:4)/Al bulk heterojunction device which resulted in a *V_oc_* of 0.64 V, a *J_sc_* of 0.00 mA/cm^2^, and a PCE of 0.27%. The overall efficiency varied with the photoactive layer thickness. Evidence obtained by photophysical measurements suggested that the efficiency for generating long-lived charge separation was substantially higher when the excited state of the organometallic polymer that preceded a photoinduced electron transfer process was a triplet state. However, the polymer absorbed light only in the blue-violet visible spectroscopic region, and consequently the efficiency was disappointing because of the low coverage of the solar spectrum.

A high photocurrent quantum yield of up to 1% was reported in 1998 by Younus *et al.* [122] for sandwich-type diode structure ITO/**MP5**/Al in air, although the phosphorescent state was not apparently involved in the photovoltaic effect. Due to the push-pull interaction between the electron-donating Pt-ethynyl group and the electron withdrawing 5,7-diphenyl-2,3-dithieno[3,4-b]-pyrazine unit, polymer **MP5** has an unusually low E_gap_ at 1.77 eV.

Wong *et al.* [123] synthesized a series of platinum(II) polyynes containing bithiazole-oligo(thienyl) rings (**MP7-10**, Figure 12). They tuned absorption, charge transport properties, and solar cell efficiency by varying the number of thienyl rings. A high PCE of up to 2.50–2.66% was obtained for **MP9** and **MP10**; the *V_oc_* was quite good and increases with the number of thiophenes, going from 0.73 to 0.88 V. Same result for short circuit current density; the *J_sc_* was considerable increased from 0.91 mA/cm^2^ to values over 6.50 mA/cm^2^. The broad EQE curves for **MP9** and **MP10** covered almost the entire visible spectrum from 350 to 700 nm with a maximum of 81.3 and 59.3%, respectively. At the same blend ratio of 1:4, the PCE increased sharply from **MP7** to **MP10** (*i.e.*, **MP7** < **MP8** < **MP9** < **MP10**), and it is remarkable that the light-harvesting ability of **MP10** could be increased by 12 times relative to **MP7** simply by adding more thienyl rings along the polymeric backbone.

Changing the bithiazole with fluorene (**MP11-14**) L. Liu *et al.* [124] obtained a similar trend, but a higher PCE (<2.9%). This was a result of a higher current density (6 times increasing), very good open circuit voltage (0.89 V) and also a very high EQE (83%).

**Figure 13.**
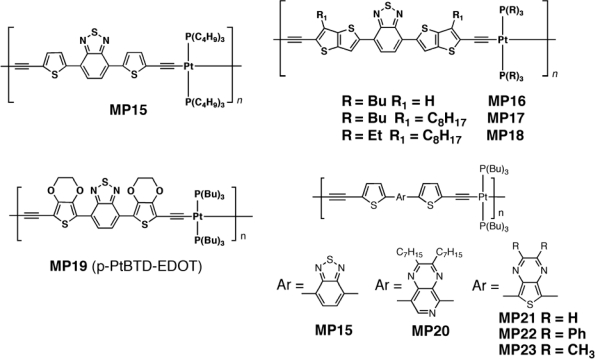
Structures of Metallopolyyne Polymers **MP15–MP23**.

The breakthrough in organometallic photovoltaics came from another work by Wong *et al.* [125]. The bulk heterojunction cells consisting in a strongly-absorbing metallopolyyne **MP15** (Figure 13) and PCBM exhibit substantial photovoltaic responses, comparable to the best reported efficiencies of fully optimized devices based on P3HT. A considerable increase in the short-circuit current density and the power-conversion efficiency was observed in **MP15** in respect of P3HT. The open-circuit voltage obtained for the best cell was 0.82 V, the short-circuit current density was 15.43 mA/cm^2^ and the fill factor was 0.39, resulting in the PCE of 4.93%. EQE as high as 87% at 570 nm was also obtained. The open-circuit voltage was higher than P3HT/PCBM cells, which was attributed to the lower HOMO level of **MP15** (−5.37 eV, compared with −5.20 eV for P3HT). The best ratio between donor and acceptor was found 1:4 wt/wt because of a better phase separation. Formation of PCBM-rich domains improved charge transport and carrier collection efficiency, which resulted in a reduction of recombination losses and in an increase in short-circuit current density. It was not a triplet state but mostly a charge-transfer excited state that contributed to the efficient photoinduced charge separation in the energy conversion for **MP15**. The charge transfer nature of the transition was also supported by solvatochromism of **MP15**. Several groups [126,127] raised serious doubts that the reported efficiencies were significantly overestimated. Baek *et al.* [128] developed a series of Pt-based polymers (**MP16**–**MP18**, Figure 13) derived from **MP15** by substituting the thiophene rings with the more rigid tienothiophene. They also changed the alkyl chain both on fused thiophene and on phosphorus. The solar cell giving the best performance was based on the highest hole mobility polymer **MP18** blended with PCBM. The device resulted in a *J_sc_* of 5.67 mA/cm^2^, an open circuit voltage of ∼0.8 V and a PCE of 2.22%. Changing the acceptor from PCBM to PC_71_BM the authors found that it was possible to increase the cell efficiency, due to the better absorbance and charge transport properties of the latter. An average power conversion efficiency of 3.73% was achieved, with a short circuit current value of 9.61 mA/cm^2^.

Very recently, Mei *et al.* [129] developed two polymers which feature a π-conjugated segment consisting of a 2,1,3-benzothiadiazole acceptor moiety flanked on either side by 2,5-thienyl donor (p-Pt-BTD-Th, **MP15**), and (3,4-ethylendioxy)-2,5-thienyl (p-PtBTD-EDOT, **MP19**) donor units, respectively (Figure 13). Both polymers absorbed strongly throughout the visible region. When tested in OPV device, if the BTD unit was flanked by (3,4-ethylenedioxy)-2,5-thienyl donors (**MP19**) a maximum PCE of 0.78%, a short circuit current of 4.56 mA/cm^2^ and a *V_oc_* of 0.50 V were achieved. Wong *et al* also reported on photovoltaic devices on **MP19** and showing lower PCE as well as *J_sc_* values, but higher *V_oc_* (0.55 V) [130]. The results achieved suggested that charge separation occurred with high internal quantum efficiency, but the overall photovoltaic performance was limited by incomplete light harvesting and low carrier mobility. The photophysical studies of the polymers revealed that, although a triplet excited state was produced following light absorption, it was too low in energy to undergo PET with PCBM. Studies carried out in solution demonstrated that quenching of the singlet state of the polymers by PCBM was efficient, thus leading to the conclusion that the photovoltaic response of the solid materials arised because of charge separation from the singlet state of the polymer.

The results pointed to the fact that, in order to harness the triplet excitons for charge separation in low-band-gap materials, it is necessary to manipulate the energy levels of either the polymer or the acceptor. If the BTD was flanked with thiophene, careful optimization of processing conditions and the active layer thickness afforded **MP15**/PCBM-based devices that exhibited peak IPCEs of 36% (*vs.* 87% at 575 nm) and overall power conversion efficiencies of 1.39%. The photovoltaic devices based on the low-band-gap polymers display considerably improved performances compared to devices based on blends of a wide-band-gap (blue-absorbing) platinum acetylide polymer.

**Figure 14.**
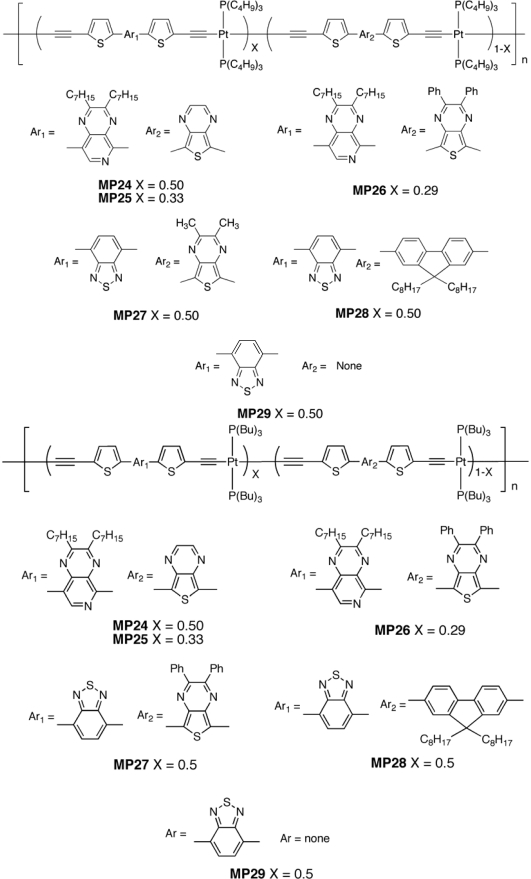
Structures of Metallopolyyne Polymers **MP24**–**MP29**.

Other examples were recently reported [131] both with PC_71_BM and PC_61_BM as acceptors. In particular, Wu *et al.* [131,132] synthesized and tested a series of Pt-containing D-A polymers and copolymers systems in bulk heterojunction solar cells (**MP15, MP20-29**). General structures are shown in Figures 13 and 14.

The authors also reported the photovoltaic device made with **MP15** with a PCE much closer to Janssen prediction (*i.e.*, 2.2%) [126]. The results are summarized in Table 1. The overall power conversion efficiency of the BHJ solar cells incorporating these organometallic “polymers as donors and PC_71_BM (or PC_61_BM) as acceptor varied widely among the polymers. The best efficiencies were 0.68%, 0.71% (**MP20** and **MP29,** respectively**)** and 2.41% for **MP15**. Open circuit voltage varied in a range from 0.3 V to 0.8 V; the short-circuit current density of the photovoltaic devices within this series of polymers was relatively low (0.17–4.21 mA/cm^2^) except in the case of **MP15** (9.65 mA/cm^2^). However, the *J*_sc_ value was much lower than the reported 13.1–15.1 mA/cm^2^ for **MP15** by Wong [125], this being the major limiting factor on the solar cell performance.

The *J*_sc_ was greatly reduced going from **MP15** to **MP20**, **MP21**, and **MP23**, for not explained reasons. Although the lower hole mobilities of **MP20** and **MP21** were considered contributing factors, however, **MP23** showed a higher mobility than **MP15** and a much lower *J*_sc_. **MP24** and **MP25** presented moderately high molecular weights but their hole mobilities were low, accounting for their rather poor *J*_sc_ values (1.39 and 0.99 mA/cm^2^). **MP22** and **MP26** were poor p-type semiconductors for BHJ solar cells as a result of their low molecular weights and poor carrier mobilities. Even though additional electron-donating groups were incorporated into copolymers **MP28** and **MP29**, their hole mobilities and *J_sc_* were not improved.

### 3.3. Donor-Fullerene Hybrids

The covalent fixation of the C_60_ group on the π-conjugated system has progressively emerged as a very active field of research [133,134]. In addition to a possible answer to the problem of phase segregation and clustering phenomena, it is also possible to provide new synthetic tools for a more global control of the interface between the π-conjugated donor and the fullerene acceptor. This should allow a fine tuning of relevant parameters such as the ratio, distance, relative orientation and mode of connection of the donor and acceptor groups. Furthermore, in addition to interesting model systems for the analysis of the fundamental aspects of photo-induced charge generation, C_60_-derivatized π-conjugated systems (**DF**) can open interesting opportunities to develop nanoscale or molecular photovoltaic devices.

This section deals with hybrids in which oligomeric/polymeric structures incorporating acetylenic linkages such as phenylene ethynylenes and thienylene ethynylenes have been attached onto C_60_, and their use as the active materials in photovoltaic devices. For details on their preparation the reader is referred to the original papers.

**Figure 15.**
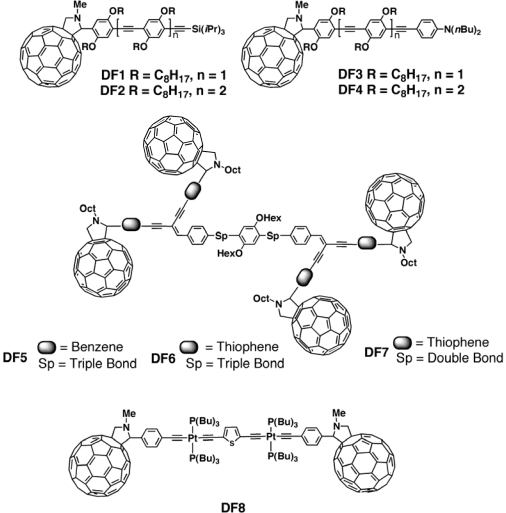
Covalently Linked Donor-Fullerene Hybrids **DF1–DF8**.

Nierengarten *et al.* [135–137] described the synthesis of the C_60_-oligophenylene ethynylene (OPE) dyads **DF1**-**DF4** (Figure 15). For all compounds the UV-vis spectrum exhibited the spectral signature of the two individual building blocks, indicating an absence of significant ground state interactions, whereas the quenching of luminescence of the π-conjugated chain clearly pointed out the occurrence of intramolecular photoinduced processes. As far as the charge carrier mobility properties are concerned, an increasing trend for the hole mobility was found in correspondence with a longer conjugated backbone. This observation was ascribed to a better stabilization of the cationic species when the length of the conjugated backbone is increased. At the same time the electron mobility, which was in all cases greater than the hole mobility, increased when the intrinsic fullerene:OPE weight ratio was higher, *i.e.*, when the oligomeric chain was shorter. The performances of the photovoltaic cells based on **DF1**–**DF4** were rather limited. The authors stated that the low mobilities measured were an important limiting factor. Indeed, having the highest average charge carrier mobility, compound **DF3** exhibited also the highest short circuit current (1.16 × 10^−6^ A/cm^2^) and the highest overall energy conversion efficiency (0.02%). Interestingly, the electron mobility of the N,N-dialkylaniline terminated derivatives **DF3** and **DF4** (3.93 × 10^−6^ cm^2^/Vs and 7.00 × 10^−7^ cm^2^/Vs, respectively) was found to be almost one order of magnitude higher when compared to the corresponding homologues **DF1** and **DF2**, leading therefore to a significantly improved performance of the corresponding devices. This was explained in terms of the different donating ability of the OPEs.

Atienza *et al.* [138] recently reported the synthesis and PV application of a light harvesting tetrafullerene nanoarray in which four C_60_ units were covalently linked to a single oligo(p-phenylene ethynylene)-type π-conjugated oligomer (**DF5**). No significant PV effect was observed for the single layer device; photophysical studies carried out in solution and in thin films demonstrated that the absence of PV activity was due to an inefficient photoinduced electron-transfer process between the OPE central core and the peripheral C_60_ units. The low tendency for photoinduced charge generation in these tetrafullerene conjugates was caused by a relatively high oxidation potential for the OPE oligomers. Nevertheless, the combination of this new conjugate with P3HT led to a device that exhibited a short-circuit current of 1.2 mA/cm^2^, a *V_oc_* of 0.65 V, and a fill factor of 0.17%. The external quantum efficiency of the devices reached 15%. This demonstrated that **DF5** acted as an efficient electron acceptor in combination with P3HT as electron donor.

More recently [139] two new tetrafullerene hybrids, **DF6** and **DF7**, were synthesized and their PV response evaluated by using them as active materials in single layer devices. In solution these nanoconjugates exhibited a very fast deactivation (∼10 ps) of the singlet excited state of the central core unit to produce both charge-separated species and C_60_ singlet excited states. The formation of a charge separate state was more pronounced for **DF7**, whose central core presents a stronger donor character. The charge-separated state recombined partially to the C_60_ centered singlet state, which underwent subsequent intersystem crossing. Photophysical studies carried out in films supported these data, demonstrating long-lived triplet excited states. For both nanohybrids, the low yield of long-lived charge carriers in thin films accounted for the limited PV response (0.005% and 0.015%, respectively).

Guo *et al.* [140] synthesized a donor-acceptor triad (**DF8**, Figure 15) consisting of a platinum-acetylide oligomer that contains fulleropyrrolidine moieties. The authors aimed at gaining insight into the mechanism, efficiency and dynamics of photoinduced charge transfer from the platinum-acetylide chromophore to a fullerene acceptor. Electrochemistry of the triad revealed four reversible redox processes. First, on the anodic sweep was observed a single, reversible oxidative wave at E_1/2_ = +0.83 V. This wave corresponds to a one-electron process and it was attributed to the oxidation of the platinum acetylide moiety. The cathodic scan reveals three, reversible waves in the potential region from −0.5 to −1.6 V. These waves represented the reduction of the two fulleropyrrolidine units. Transient absorption studies indicated that photoinduced charge transfer occurred very rapidly following photoexcitation of the platinum-acetylide chromophore with a 400 nm excitation pulse. Although a quantitative assessment of the contributions of the singlet and triplet electron transfer pathways was not possible, the authors supposed that photoinduced charge transfer occurred predominantly from the triplet state, which was produced very rapidly following photo-excitation. This means that the geminate ion radical pair which was produced by photoinduced electron transfer was in a triplet spin state. Despite the possible involvement of the triplet in the charge separation process, the charge separated state decayed rapidly. When the triad was tested in an OPV device, a short circuit current of 0.5 mA/cm^−2^, a *V_oc_* of 0.41 V and an overall power conversion efficiency of 0.056% were obtained. The peak IPCE of the device was 22% under a monochromatic light intensity of 10 μW cm^−2^. The results also demonstrated that hole as well as electron transport in the material was efficient. The good photovoltaic performance of the material was tentatively attributed to the favorable molecular packing in the solid state.

**Figure 16.**
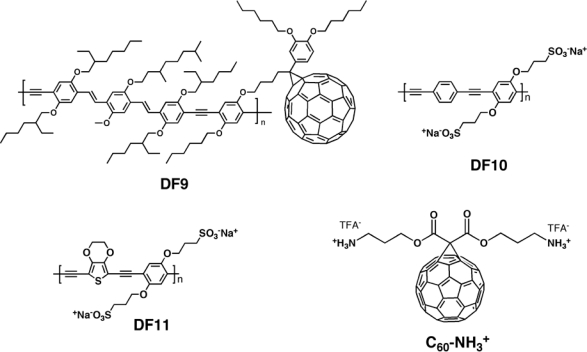
Structure of Donor-Fullerene Dyads **DF9**, **DF10**, and **DF11.**

Ramos *et al.* [141] synthesized a novel π-conjugated polymer **DF9** (Figure 16) comprising both double and triple bonds in the backbone, and carrying pendant fullerenes. Photoluminescence quenching was in thin-films of **DF9**, thus suggesting the presence of a photo-induced electron transfer between the conjugated chain and the pendant fullerene.

The solar cells based on **DF9** delivered a short circuit current of 0.42 mA/cm^2^, an open circuit voltage of 0.83 V and a fill factor of 0.29. This device exhibited peak IPCE of 6% at 480 nm. The values of the photovoltaic parameters are the highest reported so far for a cell based on a single component C_60_-π-conjugated system.

Mwaura *et al.* [142] used anionic poly-(phenylene ethynylene)-based conjugated polyelectrolytes (CPE) as electron donors (**DF10**–**DF11**), combined with a water-soluble cationic fullerene C_60_ (C_60_-NH_3_^+^) derivative as the acceptor (Figure 16) to fabricate thin-film photovoltaic cells using the layer-by-layer technique. Electrostatic layer-by-layer (LBL) deposition of polyelectrolytes has been shown to produce thin-films with molecular level thickness control. Combining absorption spectroscopy, atomic force microscopy (AFM), scanning electron microscopy (SEM) and XPS it was demonstrated that the deposited layers were spatially uniform, relatively smooth, and free of long-range phase segregation between the donor CPE and acceptor components. Indeed, the **DF9** films exhibited a more aggregated texture relative to **DF10** films, which was tentatively attributed to the **DF9**-based films being thicker, as this polymer was found to deposit more efficiently. For the optimal **DF10**/C_60_NH_3_^+^ cell, a short-circuit current of 0.50 mA/cm^2^, and an open circuit voltage of 260 mV was observed under AM 1.5 solar simulated light. Combined with a fill factor of 31%, the device gave a power conversion efficiency of ∼0.04%. The optimal photovoltaic response of **DF11**/C_60_NH_3_^+^ was not as good as that **DF10**/C_60_NH_3_^+^-based cell. Such device exhibited *V_oc_* ∼ 200 mV, *J_sc_* ∼ 0.2 mA/cm^2^, FF ∼ 25% and an overall power conversion efficiency of ∼0.01%. Visual observation of **DF11** cell revealed that the polymer bleached while it was illuminated with the high intensity AM1.5 solar light. The authors supposed that this photobleaching was partly responsible for the poor photoresponse of the cells that contain **DF11**. An interesting feature in these results was that the IPCE for the **DF9** cell fell sharply at wavelengths below 400 nm, whereas that for the **DF10** cell continued to increase for λ < 400 nm. It was demonstrated that the absorption in the region below 400 nm was mainly due to the C_60_-NH_3_^+^ component. Thus, the photoaction spectra implied that in the **DF10**/C_60_-NH_3_^+^ cells light absorbed by the C_60_-NH_3_^+^ component gives rise to photocurrent. By contrast, in the **DF9**/C_60_-NH_3_^+^ cells, light absorbed by C_60_-NH_3_^+^ seemed not to efficiently generate photocurrent. An explanation for this difference came from consideration of the thermodynamics for photo-induced charge transfer in the two cells.

Although the overall power conversion efficiencies were low, this work represents the best values yet reported on photovoltaic cells constructed using the LBL approach.

## 4. Conclusions

Since the pioneering investigations of Tang and co-workers [143], huge progresses have been made in organic solar cells and a large number of molecules and polymers were investigated. Although P3HT is still dominating OPV, (poly)arylacetylenes are an alternative and very promising class of semiconductors which have led to power conversion efficiencies which rival, in several cases, with the optimal values reported in literature.

Small-molecule approach presents attractions over polymer one in terms of tailoring structures to obtain desired features (e.g., energy levels, absorption spectra, charge carrier mobility, purity). However, in some cases, the scope of this approach is limited by the low reproducibility of the results, as the interactions within the active layer are not easily predictable. It is desirable, therefore, to achieve further progress concerning the supra-molecular control of the active layer morphology, *i.e.*, by hydrogen or halogen bonding, exploiting the rigid rod like structure of monodisperse molecules.

On the other hand, the use of polymers assures better EQEs and higher reproducibility, but it is hard to control parameters such as polydispersity. The approach based on metal-containing polyarylacetylenes seems to be really promising, because it allows to combine the interesting physical characteristics of metal complexes (e.g., electronic, optical and magnetic properties) with the solubility and processability inherent to carbon-based polymers. As widely demonstrated in Grätzel solar cells, it is also possible to fine tuning the physico-electrochemical characteristics of the organo-metallic materials.

Donor-acceptor dyads (*i.e.*, fullerene derivatives-p-type olygomers/polymers) seem to be promising as well, even though it is not yet clear what is the device limiting step (electron transfer, charge transport, *etc*) when these systems are used as active layer. More efforts are needed to address the issue.

The best results reported to date in the literature for (poly)arylacetylene- based photovoltaic devices are summarized in Table 2.

By exploiting the effect of the electron-withdrawing character of the triple bond and their rigid rod-like structure, acetylene containing materials can offer peculiar spectroelectrochemical characteristics (e.g., the ability to decrease the HOMO energy level, which is directly correlated with the tuning of open circuit voltage of the corresponding photovoltaic devices). Moreover, the aforementioned availability of efficient synthetic protocols allows the easy variation of specific structural features (e.g., the π-conjugation length, as well as the core functionalization with proper chromophoric groups), which may lead to an increase in the charge carrier mobility and photon/light harvesting, thus improving in principle both short circuit current density and fill factor. Therefore, it is desirable to explore new structures to advance their state-of-the art performance.

## Figures and Tables

**Table 1.** Summary of *J*-*V* Characteristics of D-A (Co)Polymer-based OPVs by Wu *et al.* [131].

**Polymer Blend*a***	**Thickness** (nm)	***V***_***oc***_ (V)	***J***_***sc***_ (mA/cm^2^)	**FF (%)**	**PCE** (%)
**MP15** : PC_71_BM	75	0.77	9.65	32	2.41
**MP20** : PC_71_BM	80	0.66	2.99	34	0.68
**MP21** : PC_71_BM	65	0.52	2.71	26	0.36
**MP22** : PC_71_BM	50–70	0.39	0.25	17	0.016
**MP23** : PC_71_BM	50–70	0.53	2.14	28	0.32
**MP24** : PC_61_BM	60	0.5	1.39	23	0.16
**MP25** : PC_61_BM	60	0.5	0.99	23	0.11
**MP26** : PC_61_BM	50–70	0.32	0.17	18	0.009
**MP27** : PC_71_BM	50–70	0.52	0.86	25	0.11
**MP28** : PC_71_BM	50–70	0.64	2.35	20	0.31
**MP29** : PC_71_BM	50–70	0.68	4.21	25	0.71

a 1:4 w/w.

**Table 2.** Summary of the Device Parameters for the Best (Poly)Arylacetylene-Based Devices Reported to Date in the Literature.

**Blend**	***V***_***oc***_ (V)	***J***_***sc***_ (mA/cm^2^)	**FF** (%)	**PCE (%)**
S6:PCBM [76]	0.96	2.6	45	1.17
P9:PCBM [93]	0.79	7.14	55.65	3.14
MP14:PCBM [124]	0.89	7.56	43	2.88
MP15:PCBM[125]	0.82	15.43	39	4.93
DF10:PCBM [142]	0.26	0.50	31	0.041
